# Clinical Manifestations of Non-O1 *Vibrio cholerae* Infections

**DOI:** 10.1371/journal.pone.0116904

**Published:** 2015-01-20

**Authors:** Yen-Ting Chen, Hung-Jen Tang, Chien-Ming Chao, Chih-Cheng Lai

**Affiliations:** 1 Department of Emergency Medicine, Chi Mei Medical Center, Tainan, Taiwan; 2 Department of Medicine, Chi Mei Medical Center, Tainan, Taiwan; 3 Department of Health and Nutrition, Chia Nan University of Pharmacy and Science, Tainan, Taiwan; 4 Department of Intensive Care Medicine, Chi Mei Medical Center, Liouying, Tainan, Taiwan; Beijing Institute of Microbiology and Epidemiology, CHINA

## Abstract

**Background:**

Infections caused by non-O1 *Vibrio cholera* are uncommon. The aim of our study was to investigate the clinical and microbiological characteristics of patients with non-O1 *V. cholera* infections.

**Methods:**

The clinical charts of all patients with non-O1 *V. cholera* infections and who were treated in two hospitals in Taiwan were retrospectively reviewed.

**Results:**

From July 2009 to June 2014, a total of 83 patients with non-O1 *V. cholera* infections were identified based on the databank of the bacteriology laboratories of two hospitals. The overall mean age was 53.3 years, and men comprised 53 (63.9%) of the patients. Liver cirrhosis and diabetes mellitus were the two most common underlying diseases, followed by malignancy. The most common type of infection was acute gastroenteritis (n = 45, 54.2%), followed by biliary tract infection (n = 12, 14.5%) and primary bacteremia (n = 11, 13.3%). Other types of infection, such as peritonitis (n = 5, 6.0%), skin and soft tissue infection (SSTI) (n = 5, 6.0%), urinary tract infection (n = 3, 3.6%) and pneumonia (2, 2.4%), were rare. July and June were the most common months of occurrence of *V. cholera* infections. The overall in-hospital mortality of 83 patients with *V. cholera* infections was 7.2%, but it was significantly higher for patients with primary bacteremia, hemorrhage bullae, acute kidney injury, acute respiratory failure, or admission to an ICU. Furthermore, multivariate analysis showed that in-hospital mortality was significantly associated with acute respiratory failure (odds ratio, 60.47; 95% CI, 4.79-763.90, P = 0.002).

**Conclusions:**

Non-O1 *V. cholera* infections can cause protean disease, especially in patients with risk factors and during warm-weather months. The overall mortality of 83 patients with non-O1 *V. cholera* infections was only 7.2%; however, this value varied among different types of infection.

## Introduction

*Vibrio cholerae*, gram-negative bacteria, are ubiquitous in the aquatic environment. Till now, there are more than 200 serogroups of *V. cholerae* within the species. Although *V. cholerae* O1 is the notorious strain that can cause epidemic or pandemic diarrheal disease—cholera, emerging infections due to non-O1 *V. cholerae* has become another unneglectable problem. Acute gastroenteritis is the most common clinical manifestation of non-O1 *V. cholerae* infection for both sporadic and outbreaks cases [1]. In contrast, non-O1 *V. cholerae* has been rarely involved in extra-intestinal infections, which include primary bacteremia, skin and soft tissue infections (SSTI), pneumonia, acute pyosalpinx, acute cholecystitis, endophthalmitis, peritonitis, urinary tract infection, splenic abscess, liver abscess, intracerebral abscess, meningitis, cholangitis, and empyema [2–17]. However, most of the previous studies are cases reports because of its rare occurrences. Therefore, a large-scale study about this issue is warranted to help us better understand the clinical manifestations and prognostic factors of non-O1 *V. cholerae* infections.

As we know, *V. cholerae* infections is strongly associated with water exposure or ingestion of raw food [2, 3]. The coastal area in southern Taiwan, located in subtropical area, is the kind of environment suitable for the growth of *V. cholerae.* Taking advantage of the geographic characteristics, we conduct this retrospective study to investigate the clinical manifestations, microbiological characteristics, response to treatment, and outcome in a medical center and a regional hospital in southern Taiwan.

## Materials and Methods

### Hospital setting and patient selection

This study design is a retrospective study and was conducted at two hospitals, Chi Mei Medical Center, a 1290-bed referral medical center, and Chi Mei Medical Center, Liouying branch, a 900-bed regional hospital, located in Tainan, Taiwan. From the computerized database of the bacteriology laboratory from July 2009 to June 2014, patients whose cultures yielded non-O1 *V. cholerae* species were identified. Because this study only focused on V. cholerae infection, polymicrobial infections were excluded. The clinical charts of all patients included in this study were retrospectively reviewed. Information regarding age, gender, underlying immunocompromising conditions including history of immunosuppressant drug use, diabetes mellitus, liver cirrhosis, chronic kidney disease, and malignancy were collected. The data was collected on a routine basis and the analysis was carried out retrospectively. The records and information of patients were anonymized and de-identified prior to analysis. Therefore, no informed consent was required and it was specifically waived by Institution Review Board. Ethics approval was obtained from Institution Review Board of Chi Mei Medical Center.

### Bacterial isolates

*V. cholerae* by conventional identification methods and two commercial systems, including the API 20E system (bioMerieux, Vitek, Inc., Hazelwood, MO, USA) and the Phoenix system (NMIC/ID-72, Becton Dickinson, Sparks, MD). All of the clinical isolates were identified as *V. cholerae* non-serogroup O1 based on negative reaction by slide agglutination with polyvalent O1 (Difco, Becton Dickinson, Sparks, MD). However, *V. cholerae* serogroup O139 was not tested due to lack of reagents.

### Definitions

The diagnosis of the bacteremia infection focus was made based on clinical, and bacteriological investigations. Acute gastroenteritis was classified as clinical diarrhea plus a positive stool culture. SSTI was defined as clinical soft tissue inflammation plus positive soft tissue or pus culture. Peritonitis was diagnosed in patients with clinical peritoneum inflammation plus positive culture of ascites. Biliary tract infections were diagnosed in patients with clinical hepatobiliary tract inflammation plus positive bile cultures. The bile specimens were collected by percutaneous transhepatic cholangiodrainage, percutaneous gallbladder drainage, or operation. Pneumonia was defined as a positive culture for *V. cholerae* in purulent sputum samples and the presence of newly developed lung infiltrates. Genitourinary tract infection (UTI) was defined as positive urine culture with growth of 10^5^ CFU/ml and pyuria. If no primary focus could be identified, the bacteremia was classified as primary. Shock was diagnosed in patients with a systolic blood pressure < 90 mm Hg or in patients who required inotropic agents to maintain blood pressure. In-hospital mortality was defined as death from all causes during the study episode of hospitalization.

### Statistical analysis

Data were analyzed using SPSS version 11.0 software. Continuous variables were expressed as mean ± standard deviation. Comparisons between continuous variables were analyzed using the Wilcoxon rank sum test or Student’s independent *t* test, as appropriate. Comparisons between or among categorical variables were made using the chi-square or Fisher’s exact test. The analyses of risk factors and outcomes were performed using the chi-square test. A multivariable forward logistic regression model was used to identify risk factors for mortality. Statistical significance was set at *P* < 0.05.

## Results

### Clinical characteristics

During the study period, a total of 83 patients with non-O1 *V. cholerae* infections were identified based on the databank of the bacteriology laboratories of two hospitals. The clinical characteristics of all patients with non-O1 *V. cholerae* infections are summarized in Table 1. The overall mean age was 53.3 years, and men comprised 53 (63.9%). Liver cirrhosis and diabetes mellitus were the two most common underlying diseases, followed by malignancy. Among 20 patients with liver cirrhosis, 10 cases had history of alcoholism, and 8 patients had ascites. Hepatocellular carcinoma was the most common type of cancer (n = 7), followed by pancreatic cancer (n = 3). Abdominal pain (n = 60, 72.3%) was the most common presentation, followed by diarrhea (n = 52, 62.7%), and fever (n = 34, 41.0%). Additionally, initial shock and the presence of hemorrhagic bullae were noted in 9 (10.8%) and 5 (6.0%) patients, respectively. The most common type of infection was acute gastroenteritis (n = 45, 54.2%), followed by biliary tract infection (n = 12, 14.5%) and primary bacteremia (n = 11, 13.3%). Other types of infection were rare, such as peritonitis (n = 5, 6.0%), SSTI (n = 5, 6.0%), urinary tract infection (n = 3, 3.6%) and pneumonia (2, 2.4%). Secondary *V. cholerae* bacteremia was noted in 22 episodes of various type of infections, including acute gastroenteritis (n = 7), biliary tract infection (n = 7), peritonitis (n = 5), SSTI (n = 2), and urinary tract infection (n = 1). Among 45 patients with acute gastroenteritis, three had initial shock. However, none of them had blood stool or resemble dysentery. 3 of five patients with SSTI had presentation of necrotizing fasciitis, and all of them had underlying conditions, such as liver cirrhosis, diabetes mellitus, and chronic kidney disease. Only one had concomitant *V. cholerae* bacteremia and in-hospital mortality.

**Table 1:** Demographic characteristic of 83 patients with non-O1 *Vibrio cholerae* infections.

**Characteristic**	**No. (%) of all patients (n = 83)**	**No. (%) of patients with acute gastroenteritis (n = 45)**	**No. (%) of patients with biliary tract infections (n = 12)**	**No. (%) of patients with primary bacteremia (n = 11)**
Age, mean ± SD (years)	53.3 ± 19.3	45.3 ± 17.0* ^,^ ^†^	66.5 ± 16.7*	61.0 ± 14.1^†^
Male (%)	53 (63.9)	23 (51.1)^†^	10 (83.7)	10 (90.9)^†^
Underlying conditions				
Liver cirrhosis	20 (24.1)	6 (13.3)^†^	1 (8.3)^＃^	8 (72.7)^†^ ^,^ ^＃^
Diabetes mellitus	19 (22.9)	8 (17.8)^†^	2 (16.7)	6 (54.5)^†^
Cancer	15 (18.1)	2 (4.4)	2 (16.7)	1 (9.1)
Hepatitis B infection	10 (12.0)	2 (4.4)	0 (0.0)	3 (27.3)
Hepatitis C infection	10 (12.0)	4 (8.9)	2 (16.7)	4 (36.4)
Chronic kidney disease	7 (8.4)	2 (4.4)	0 (0.0)	1 (9.1)
Immunosuppressant use	3 (3.6)	1 (2.2)	1 (8.3)	0 (0.0)
Steroid use	4 (4.8)	1 (2.2)	1 (8.3)	0 (0.0)
Alcoholism	14 (16.9)	5 (11.1)	2 (16.7)	4 (36.4)
Initial presentation				
Abdominal pain	60 (72.3)	37 (82.2)	10 (83.3)	6 (54.5)
Diarrhea	52 (62.7)	45 (100.0)* ^,^ ^†^	2 (66.7)*	2 (18.2)^†^
Fever	34 (41.0)	11 (24.4)^†^	34 (41.0)	8 (72.7)^†^
Shock	9 (10.8)	3 (6.7)	0 (0.0)	2 (18.2)
Hemorrhagic bullae	5 (6.0)	0 (0.0)^†^	0 (0.0)	2 (18.2)^†^
Laboratory examinations				
White blood cell	12116.4 ± 9661.1	13154.3 ± 11159.8	15833.3 ± 10879.9	7727.3 ± 3596.4
Hemoglobin	12.7 ± 3.6	13.9 ± 4.3^†^	13.7 ± 1.9^＃^	10.6 ± 2.0^†^ ^,^ ^＃^
Creatinine	1.7 ± 2.0	1.9 ± 2.8	1.2 ± 0.4	1.6 ± 0.5
Glutamic oxaloacetate transaminase	127.9 ± 256.3	47.4 ± 38.6* ^,^ ^†^	286.8± 498.0	155.5 ± 244.2^†^
Total bilirubin	3.7 ± 3.2	2.4 ± 2.1	3.3 ± 2.4	4.4 ± 3.5
C-reactive protein	38.8 ± 62.3	11.8 ± 16.1* ^,^ ^†^	75.2 ± 92.9*	61.2 ± 66.4^†^
Concomitant bacteremia	33 (39.8)	7 (15.6)*	7 (58.3)* ^,^ ^＃^	11 (100.0)^†^ ^,^ ^＃^
Intensive care unit admission	14 (16.9)	3 (6.7)^†^	0 (0.0)^＃^	6 (54.5)^†^ ^,^ ^＃^
Acute respiratory failure	9 (10.8)	3 (6.7)	0 (0.0)	3 (27.3)
Acute kidney injury	15 (18.1)	5 (11.1)	2 (16.7)	4 (36.4)
In-hospital mortality	6 (7.2)	1 (2.2)^†^	0 (0.0)	3 (27.3)^†^

*Significant difference between acute gastroenteritis and biliary tract infection

^†^ Significant difference between acute gastroenteritis and primary bacteremia

^＃^Significant difference between biliary tract infection and primary bacteremia

**Figure 1:**
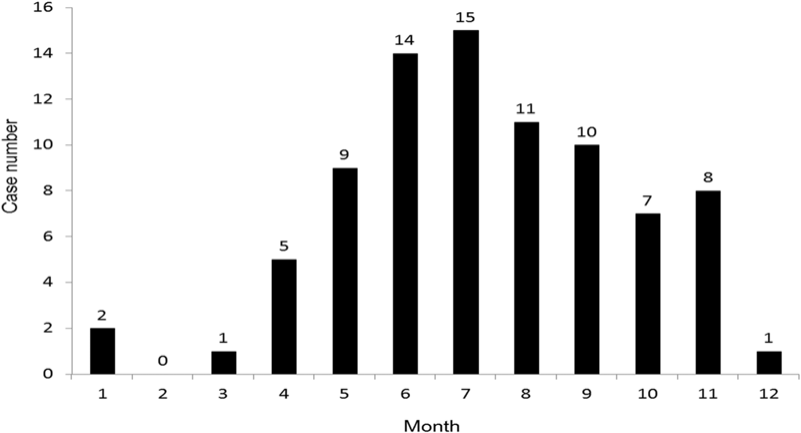
Distribution of non-O1 *Vibrio cholerae* infections by month.

July and June were the most common months of occurrence of *V. cholerae* infections, followed by August, September, and May (Fig. 1). The patients with acute gastroenteritis was significant younger than the patients with biliary tract infection and primary bacteremia. The patients with primary bacteremia had more liver cirrhosis than the patients with acute gastroenteritis and biliary tract infection. Fever was more common among the patients with primary bacteremia than with acute gastroenteritis. C-reactive protein was significant higher among the patients with biliary tract infection and primary bacteremia than the patients with acute gastroenteritis. Three mortalities developed in patients with primary bacteremia, and each one developed in patient with SSTI, peritonitis, and acute gastroenteritis. The patients with primary bacteremia had higher in-hospital mortality than the patients with biliary tract infections.

### Comparison between patients with mortality and survival

Fifteen (18.1%) and 9 (10.8%) patients had the complication as acute kidney injury requiring emergent hemodialysis and acute respiratory failure requiring mechanical ventilator, respectively. Overall, 14 patients required intensive care unit admission, and in-hospital mortality was 7.2%. Analysis of risk factor for mortality among 83 patients with *V. cholerae* infections was shown in table 2. The in-hospital mortality was significantly associated with the presence of hemorrhage bullae, acute kidney injury, acute respiratory failure, and admission to ICU. However, a multivariable analysis showed that the in-hospital mortality was only significantly associated with acute respiratory failure (odds ratio, 60.47; 95% CI, 4.79–763.90, P = 0.002).

**Table 2:** Comparison between patients with survival and mortality.

	**No (%) of patients with survival (n = 77)**	**No (%) of patients with mortality (n = 6)**	**Univariable**			**Multivariable**		
			**Odds ratio**	**P value**	**95% CI**	**Odds ratio**	**P value**	**95% CI**
Fever	31 (40.3)	3 (50.0)	1.484	0.685	0.281–7.834			
Shock on presentation	7 (9.1)	2 (33.3)	5.000	0.13	0.773–32.336			
Hemorrhagic bullae	2 (2.6)	3 (50.0)	**37.500**	**0.002**	**4.461–315.263**	15.971	0.082	0.703–363.022
Diabetes mellitus	17 (22.1)	2 (33.3)	1.765	0.61	0.297–10.472			
Liver cirrhosis	17 (22.1)	3 (50.0)	3.529	0.15	0.652–19.099			
Chronic kidney disease	7 (9.1)	0 (0.0)	0.909	1.00	0.847–0.976			
Cancer	14 (18.2)	1 (16.7)	1.125	1.00	0.117–10.852			
Concomitant bacteremia	28 (36.4)	5 (83.3)	**8.750**	**0.034**	**0.973–78.706**			
Acute respiratory failure	4 (5.2)	5 (83.3)	**91.250**	**<0.001**	**8.520–977.340**	**60.469**	**0.002**	**4.790–763.90**
Acute kidney injury	10 (13.0)	5 (83.3)	**33.5**	**0.001**	**3.540–317.044**			
ICU admission	9 (11.7)	5 (83.3)	**37.778**	**<0.001**	**3.955–360.865**			

## Discussion

As toxigenic *V. cholerae* draws the most attention of *V. cholerae* infections, the clinical data for non-O1 *V. cholerae* infections are limited. During this 5-year survey, a total of 83 patients with non-O1 *V. cholerae* infections were identified from two hospitals in southern Taiwan. To the best of our knowledge, this study is the largest series of patients with non-O1 *V. cholerae* infections, and we have several significant findings. In this study, we found the clinical manifestations of non-O1 *V. cholerae* infections are various. In addition to the most common presentation—acute gastroenteritis, non-O1 *V. cholerae* infections can presented as biliary tract infection, primary bacteremia, peritonitis, SSTI, urinary tract infection and pneumonia. Moreover, we noted that among the different types of clinical infections, the characteristics of the infected patients differed accordingly. For example, patients with biliary tract infections and primary bacteremia had greater rates of diabetes mellitus, and liver cirrhosis, malignancy, and higher level of C-reactive protein than patients with acute gastroenteritis. This is consistent with previous studies showing that persons with known liver disease, and diabetes mellitus, are at high risk of invasive *Vibrio* infections [18–21]. Therefore, clinicians should consider non-O1 *V. cholerae* as etiologic agents in cases of invasive infections in persons with liver cirrhosis and diabetes mellitus.

In the present work, the overall mortality rate of patients with non-O1 *V. cholerae* infections was only 7.2% (n = 6). For each type of infection, the mortality was 27.3% (3/11), 20% (1/5), 20% (1/5) and 7.2% (6/83) among of patients with primary bacteremia, SSTI, peritonitis, and acute gastroenteritis, respectively. Furthermore, the mortality rate of patients with primary bacteremia was significant higher than patients with acute gastroenteritis (p < 0.05). By univariable analysis, we found that the presence of hemorrhagic bullae, concomitant bacteremia, acute kidney injury, acute respiratory failure and ICU admission were poor prognostic factors. Moreover, by multivariable analysis, acute respiratory failure was found to be the only independent and significant risk factors associated with mortality. Although the impact of hemorrhagic bullae on mortality did not reach statistical significance (p = 0.082) by multivariable analysis, it may be caused by limited cases number. However, the trend should be evident; therefore, clinicians should be alert about the presence of hemorrhage bullae as the risk factor of poor outcome.

In this study, we also assess the association between seasonality and non-O1 *V. cholerae* infections. We found that June and July are the most common months of non-O1 *V. cholerae* infections, and about 80% of cases occur between May and October. It indicates the strong relationship between warm weather and the occurrence of non-O1 *V. cholerae* infections. As previous report [2], physicians should consider this pathogen as possible infectious agent for patients with risk factor, especially in the warm weather.

This study had several limitations. First, we did not determine the in vitro antibiotic susceptibility profiles of the *Vibrio* species. Therefore, we cannot evaluate the association between antimicrobial agent and outcome. Second, this study is a retrospective investigation that may suffer from sources of bias, such missing data. We cannot obtain the exposure history of aquatic environment and seafood. Third, we used all-cause mortality for outcome analysis and did not evaluate the mortality attributable to non-O1 *V. cholerae* infections. Forth, both the biochemical based test used in this study may wrongly identify some of the other vibrios/aeromonads as *V. cholerae*., and molecular based identification should be adopted for confirmation of *V cholerae* isolates. In addition, analysis of virulence gene properties of *V. cholerae* isolated is important, especially the strains associated with diarrhea. In our country, cholera is a notifiable disease. If clinicians suspect that the *V. cholera* infection may be cholera, they need to report the case to Taiwan CDC and send the strain for further confirmatory microbiology study. Both of two hospitals in the present work should follow this policy, therefore, all of the cases in this study are supposed not to be toxigenic strain despite we did not keep clinical isolate for further molecular-method based confirmation test and analysis of virulence gene. Finally, the cases number remains limited and further large-scale study is warranted to better understand the clinical manifestations of non-O1 *V. cholerae* infections.

In conclusion, non-O1 *V. cholerae* infections are not uncommon pathogens that can cause protean disease, especially for patients with risk factors and in warm weather months. The overall mortality is only 7.2%; however, it varies among different type of infection. Moreover, the mortality is significant associated with the presence of hemorrhagic bullae, concomitant bacteremia, acute kidney injury, acute respiratory failure and ICU admission by univariable analysis and, acute respiratory failure by multivariable analysis.
